# Determinants of duck Tembusu virus NS2A/2B polyprotein procession attenuated viral replication and proliferation in vitro

**DOI:** 10.1038/s41598-020-68271-0

**Published:** 2020-07-24

**Authors:** Bowen Jiang, Wei Zhang, Yuanyuan Wu, Tao Wang, Mingshu Wang, Renyong Jia, Dekang Zhu, Mafeng Liu, Xinxin Zhao, Qiao Yang, Ying Wu, ShaQiu Zhang, YunYa Liu, Ling Zhang, YanLing Yu, Leichang Pan, Shun Chen, Anchun Cheng

**Affiliations:** 1 Research Center of Avian Disease, College of Veterinary Medicine, Sichuan Agricultural University, Chengdu, 611130 Sichuan China; 2 Institute of Preventive Veterinary Medicine, Sichuan Agricultural University, Chengdu, 611130 Sichuan China; 3 Key Laboratory of Animal Disease and Human Health of Sichuan Province, Chengdu, 611130 Sichuan China

**Keywords:** Virology, Retrovirus

## Abstract

Duck Tembusu virus (DTMUV), a mosquito-borne Flavivirus, has caused serious economic losses for the Chinese poultry industry. The genome is translated into a polyprotein that is cleaved to mature protein by host and viral proteases in the host cell, and this proteolytic process is important for the viral life cycle. However, the cleavage mechanism of DTMUV polyprotein is still unclear. In this study, we identified that several amino acids (P1-R, P1′-G, P2-R, P3-T, and P4-V) were vital for NS2A/2B cleavage. Meanwhile, both NS2A and NS2B were essential *in cis* for polyprotein NS2A/2B intramolecular cleavage. Subsequently, a DTMUV replicon and an infectious clone showed that the P1 site is essential to viral replication, while a mutation in P1′ could boost viral RNA replication. Furthermore, a recombinant virus with P1 and P1′ site mutations named rDTMUV-NS2A/2B-P1P1′(AA) was rescued from transfected BHK21 cells. The maximum viral titers and viral genome copies of rDTMUV-NS2A/2B-P1P1′(AA) were much lower than those of rDTMUV-WT both in the intracellular and extracellular samples of transfected and infected BHK21 cells. Taken together, the NS2A/2B cleavage sites processed by the NS2B3 protease are vital for DTMUV proliferation and virulence.

## Introduction

Duck Tembusu virus (DTMUV) belongs to the Flavivirus genus, which includes many medically important viruses, such as Japanese encephalitis virus (JEV), dengue virus (DENV), tick-borne encephalitis virus (TBKV), and zika virus that can cause serious diseases in humans. In recent years, DTMUV outbreaks in China have caused serious economic losses^1,2^. DTMUV was first isolated in 1955 from *Culex tritaeniorhynchus* mosquitoes in Kuala Lumpur, Malaysia and Thailand^3,4^. DTMUV causes egg-drop syndrome in waterfowl, which was first reported in 2011, and the main pathological features observed are ovarian hyperemia, hemorrhage, degeneration, distortion, macrophage and lymphocyte infiltration, and hyperplasia^1,2,5^. As reported, DTMUV was lethal in mice following intracerebral inoculation and grew well in many mammalian cells^6,7^. Thus, DTMUV has a potential impact on public health.

**Figure 1:**
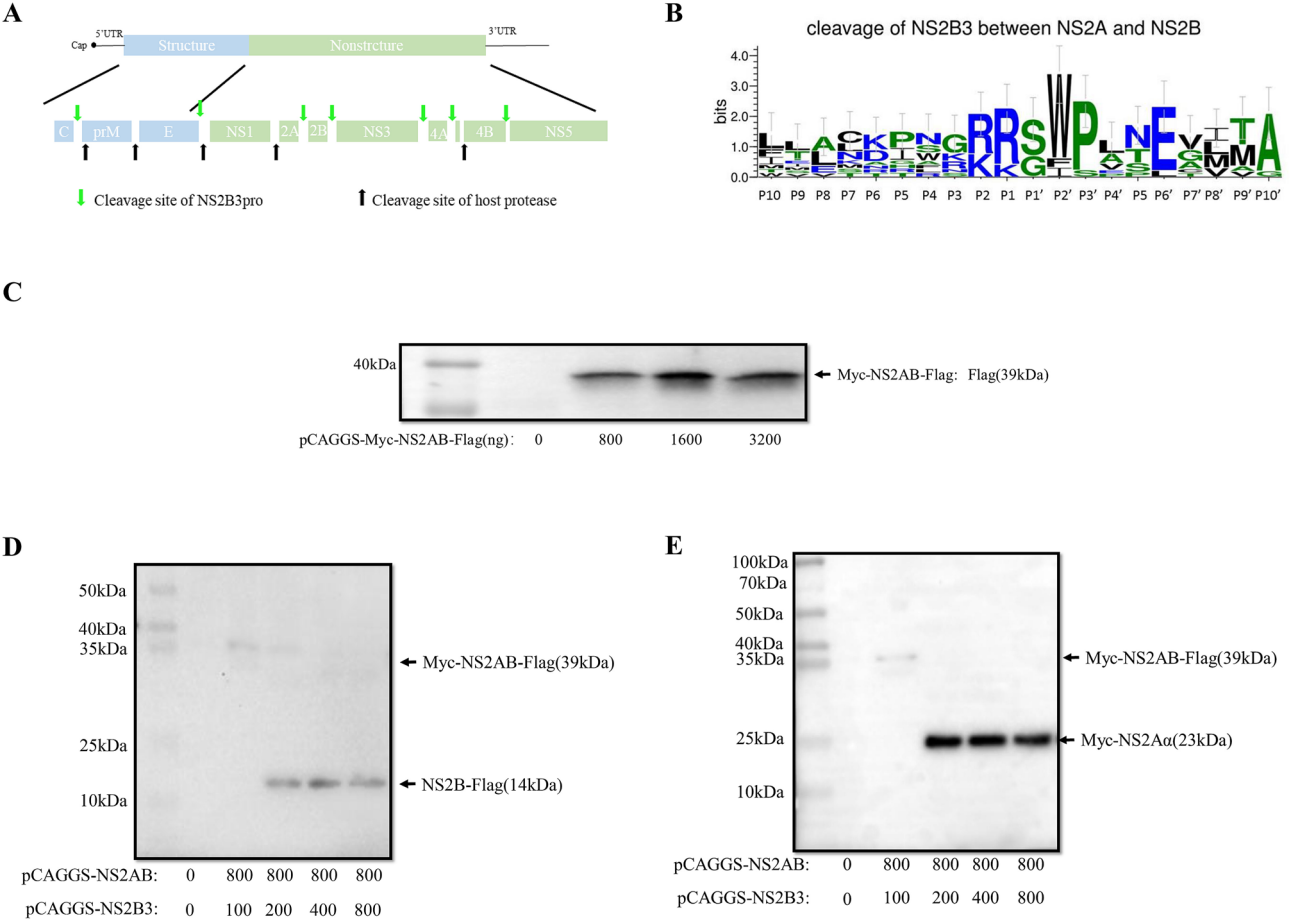
Cleavage of NS2A/2B by NS2B3. (**A**) Genome structure and cleavage sites of flavivirus polyprotein. (**B**) Conservative amino acid residues near the NS2A/2B cleavage site by comparison of different flaviviruses, including DENV, JEV WNV, YFV, TBEV, BGAV, ZIKA and KUN. (**C**) Overexpression of DTMUV NS2A/2B in transfected DEFs. DEF cells were transfected with different concentrations of pCAGGS-Myc-NS2A/2B-Flag and the cells were harvested 24 h post transfection. (**D**, **E**) Cleavage of NS2A/2B by NS2B3. DEF cells were cotransfected with plasmids expressing NS2A/2B and with different concentrations of NS2B3 plasmids, and proteins of interest were detected by WB 24 h post transfection. (**D**) Mouse anti-Flag monoclonal antibody was used as the primary antibodies, (**E**) Mouse anti-Myc monoclonal antibody was used as the primary antibody.

Flaviviruses possess a single-stranded positive-sense RNA genome, that encodes one polyprotein^2,8^. When flaviviruses infect cells, the viral genome is first translated into a polyprotein, then the polyprotein is cleaved by host protease and a viral protease (NS2B3) in endoplasmic reticulum (ER) to form ten functional proteins (Fig. 1A) including three structural proteins (C, prM and E) and seven nonstructural proteins (NS1, NS2A, NS2B, NS3, NS4A, NS4B, and NS5).The NS3 protease can cleave the polyprotein with its cofactor NS2B^9–11^. The N-terminal 180 amino acids possess protease activity, and as previously escribed, the flavivirus NS2B3 protease can cleave 6 sites in the viral polyproteins C/prM, NS2A/2B, NS2B/3, NS3/4A, NS4A/2K, and NS4B/5 (Fig. 1A)^12,13^. All of these cleavages are necessary for the virus life cycle, for example, NS2A/2B (39 kDa) can be cleaved to NS2A(25 kDa) and NS2B(14 kDa), then NS2A can be cleaved to NS2Aα, which is important to the assemble of virus^14^.

As mentioned before, the substrate of flavivirus NS2B3pro possesses a similar characteristic, where two sites (P1 and P2) before the junction are often occupied by basic amino acids and the site behind the junction (P1′) is often occupied by a short-chain amino acid^15^, such as Ala, Gly and Trp (Fig. 1B). In addition, previous reports have shown that the conformation of the substrate is very important for cleavage^16^. Not only the amino acids located on both sides of the junction but also the proteins spanning the cleavage sites are important determinants of processing. However, the cleavage of the DTMUV polyprotein by NS2B3 has not been described.

In our study, we aimed to clarify the proteinase cleavage sites of NS2A and NS2B, as well as determine amino acid requirements *in trans* for NS2B3 processing. Moreover, the role of the intramolecular proteinase cleavage sites between NS2A (25 kDa) and NS2B (14 kDa) in the proliferation of duck Tembusu virus was studied. We found that the amino acids near the cleavage sites showed differential effects on NS2A/2B cleavage. Moreover, both the NS2A and NS2B proteins were required *in cis* for NS2A/2B proteolytic processing, while NS2Aα intermolecular proteinase cleavage was NS2B independent. Subsequently, the effects of the cleavage sites on viral RNA replication and proliferation were studied in detail by using a DTMUV replicon and an infectious clone. Our study shed slight on the correlation between NS2A/2B polyprotein processing and virus virulence.

## Results

### DTMUV NS2A/2B can be cleaved by NS2B3

A previous study demonstrated that DENV NS2A/2B can be cleaved by DENV NS2B3^17^. However, there is no study has proven that DTMUV NS2A/2B can be cleaved by its NS2B3, so in our study, we demonstrated that firstly. After the plasmid pCAGGS-Myc-NS2A/2B-Flag was constructed successfully, we verified the expression of NS2A/2B in DEFs at different plasmid concentrations, and the protein was detected by Western blot, as shown in Fig. 1C. Then, pCAGGS-Myc-NS2A/2B-Flag and different volume of pCAGGS-NS2B3-His were cotransfected to DEFs, and the cells were harvested at 24 h post transfection and detected by western blot. DTMUV NS2A/2B could be cleaved even at a low dose of NS2B3, and when 200 ng pCAGGS-NS2B3-His plasmid was cotransfected with 800 ng pCAGGS-Myc-NS2A/2B-Flag plasmid, NS2A/2B was completely cleaved (Fig. 1D, E).

### Both NS2A and NS2B are *in cis* needed for NS2A/2B cleavage, and DTMUV NS2B3pro can cleave NS2A

**Figure 2:**
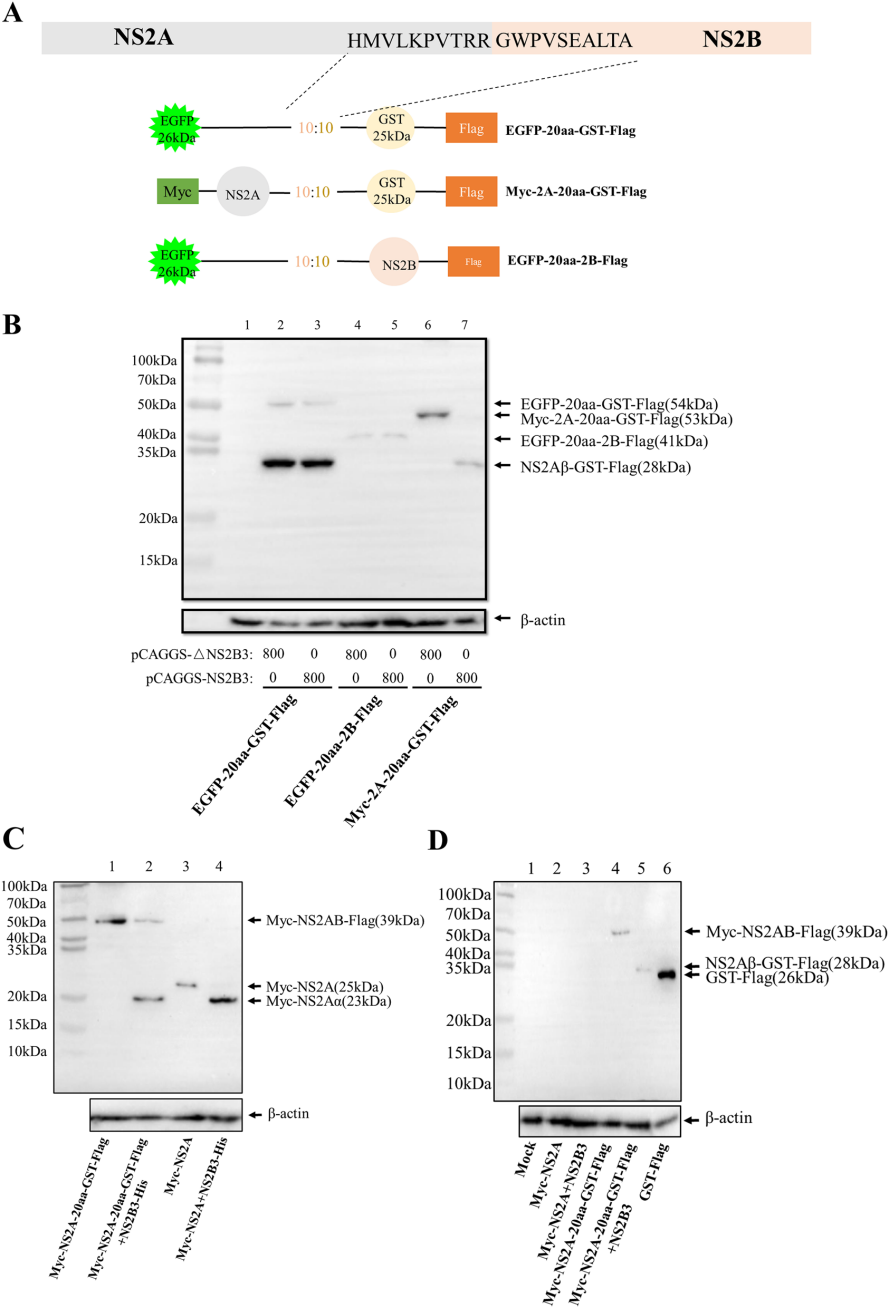
Both NS2A and NS2B are *in cis* needed for NS2A/2B cleavage, while DTMUV NS2B3pro can cleave NS2A. (**A**) Schematic of three different constructs. NS2A or NS2B were replaced by EGFP or GST, respectively, and the two were linked by 20 amino acids. (**B**) The constructs were coexpressed with NS2B3 in DEFs and harvested 24 h post transfection for WB analysis. Mouse anti-Flag monoclonal antibody was used as the primary antibody. Line 1: mock cells transfected with empty vector; line 2: cells transfected with EGFP-20aa-GST and ∆NS2B3; line3: cells transfected with EGFP-20aa-GST and NS2B3; line 4: cells transfected with EGFP-20aa-NS2B and ∆NS2B3; line 5: cells transfected with EGFP-20aa-NS2Band NS2B3; line 6: cells transfected with NS2A-20aa-GST and ∆NS2B3; line 7: cells transfected with NS2A-20aa-GSTand NS2B3. (**C**) DEFs were transfected with different construct plasmids and harvested 24 h post transfection for WB analysis. Mouse anti-Myc monoclonal antibody was used as the primary antibody. Line 1: DEF cells were transfected with NS2A-20aa-GST; line 2: DEF cells were transfected with NS2A-20aa-GST and NS2B3; line 3: DEF cells were transfected with NS2A; line 4: DEF cells were transfected with NS2A and NS2B3. (**D**) DEFs were transfected with different construct plasmids and harvested 24 h post transfection for WB analysis. Mouse anti-Flag monoclonal antibody was used as the primary antibody. The GST plasmid was used as a positive control to evaluate the molecular weight of the cleaved proteins (NS2Aβ-GST). Line 1: mock cells were transfected with empty vector; Line 2: DEF cells were transfected with NS2A; line 3: DEF cells were transfected with NS2A and NS2B3; line 4: DEF cells were transfected with NS2A-20aa-GST; line 5: DEF cells were transfected with NS2A-20aa-GST and NS2B3; and line 6: DEF cells were transfected with GST-Flag.

To determine whether NS2A or NS2B is needed *in cis* for its cleavage, we constructed plasmids expressing NS2A-20aa-GST, EGFP-20aa-NS2B, and EGFP-20aa-GST (Fig. 2A). Each of the plasmids was cotransfected with NS2B3pro to verify its cleavage by NS2B3pro. The results showed that none of the recombinant proteins could be cleaved by NS2B3pro, excluding NS2A-20aa-GST (Fig. 2B). It has been shown that YFV NS2B3 can cleave NS2A at the sequence QK/T to form NS2Aα^23^. To determine whether NS2A is needed *in cis* for NS2A/2B cleavage, we performed the following study. An NS2A eukaryotic expression plasmid was constructed and named Myc-NS2A. Surprisingly, we found that NS2A could be cleaved by NS2B3 when using Myc-NS2A as a positive control. As shown in Fig. 2C, a protein band smaller than NS2A was detected, and was assumed to be NS2Aɑ. Moreover, a protein band larger than GST (GST-Flag as positive control) was detected and was assumed to be NS2Aβ (Fig. 2D). This further indicates that NS2A-20aa-GST can be cleaved by NS2B3 at the NS2Aα cleavage sites but not at the site between NS2A and NS2B. Overall, DTMUV NS2B3pro can cleave NS2A/2B into NS2Aα, NS2A, and NS2B, while both NS2A and NS2B were needed *in cis* for NS2A/2B cleavage.

### The P2-P4 amino acid sites of DTMUV NS2A/2B differentially affectNS2B3 proteolytic processing

**Figure 3:**
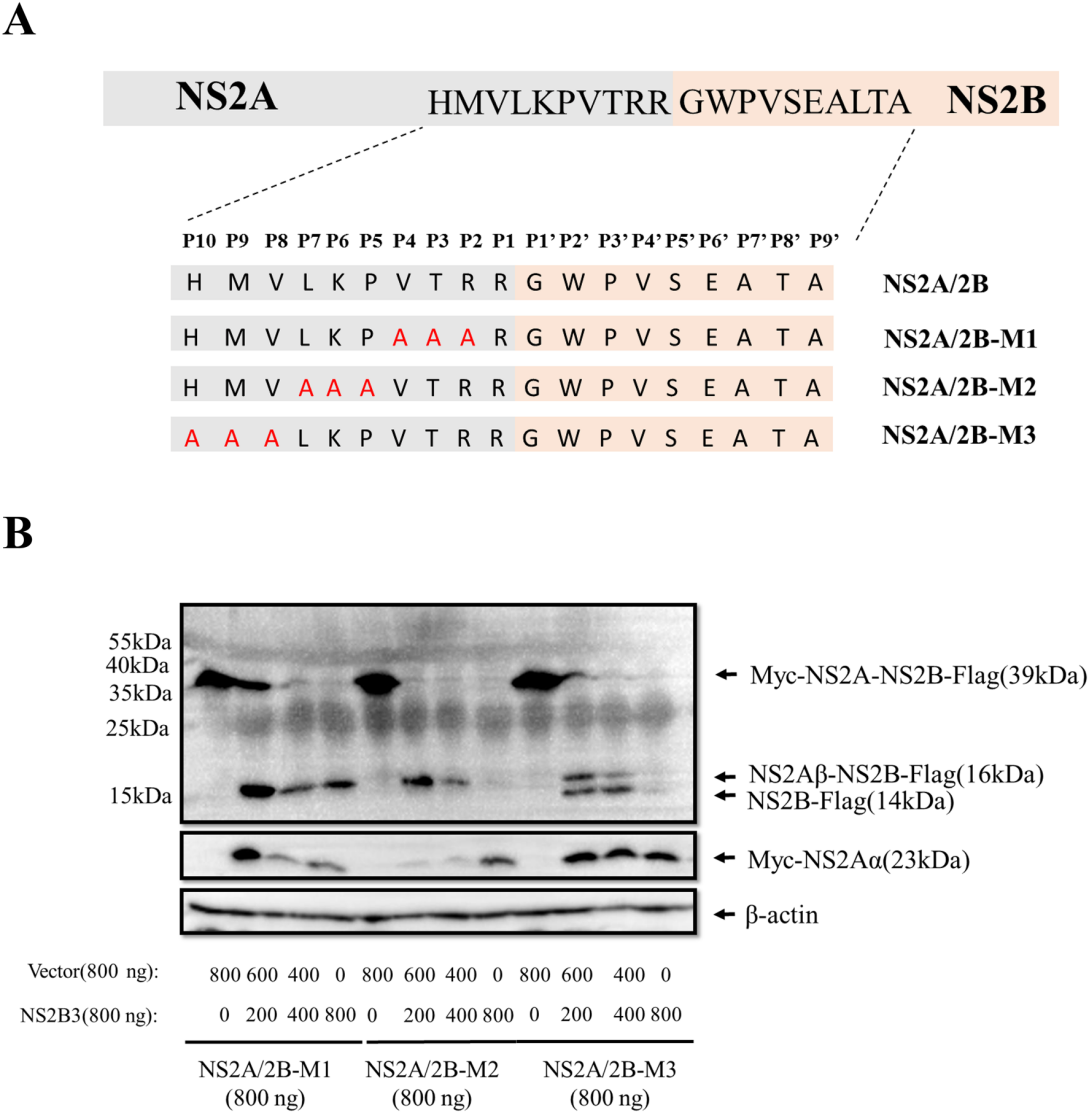
The P2-P4 amino acid sites of DTMUV NS2A/2B differentially affect NS2B3 proteolytic processing. (**A**) Schematic of the construction of the triple Ala mutation NS2A/2B-M plasmid. NS2A/2B-M1: the P2, P3 and P4 sites were mutated to Ala; NS2A/2B-M2: the P5, P6 and P7 sites were mutated to Ala; NS2A/2B-M3: the P8, P9 and P10 sites were mutated to Ala. (**B**) DEFs were cotransfected with NS2A/2B-M and different concentrations of NS2B3, and the cells were harvested 24 h post transfection for WB. Mouse anti-Myc mAb and anti-Flag mAb were used as the primary antibodies.

To determine whether P2-P10 amino acid are needed for its cleavage, the P2-P10 amino acid sites of DTMUV NS2A/2B were mutated into Ala as shown in Fig. 3A. The following plasmids were constructed: NS2A/2B-M1(P2-P4, RTV-AAA), NS2A/2B-M2(P5-P7, PKL-AAA), and NS2A/2B-M3(P8-P10, VMH-AAA). Each mutated recombination plasmid was cotransfected with NS2B3 in DEFs. As expected, NS2A/2B-M1 could not be processed by NS2B3 without forming NS2B (Fig. 3B). This finding suggested that the P2-P4amino acids were required for NS2B3 processing. NS2A/2B-M2 was incompletely processed by NS2B3 without forming NS2B. NS2A/2B-M3 was partly processed by NS2B3, forming NS2Aɑ, NS2Aβ-NS2B and NS2B (Fig. 3B), which indicates that the P5-P10 sites play a role in the process but that these sites were not determining factors. Additionally, the P5-P10 site mutations only showed mild effects on NS2B3 proteolytic processing. Collectively, our data indicated that the P2, P3 and P4 sites of NS2A/2B are very important for its cleavage, while P5-P10 have some influence on proteolytic processing.

### The P1 and P1′ amino acid sites of NS2A/2B are vital for NS2B3 proteolytic processing

**Figure 4:**
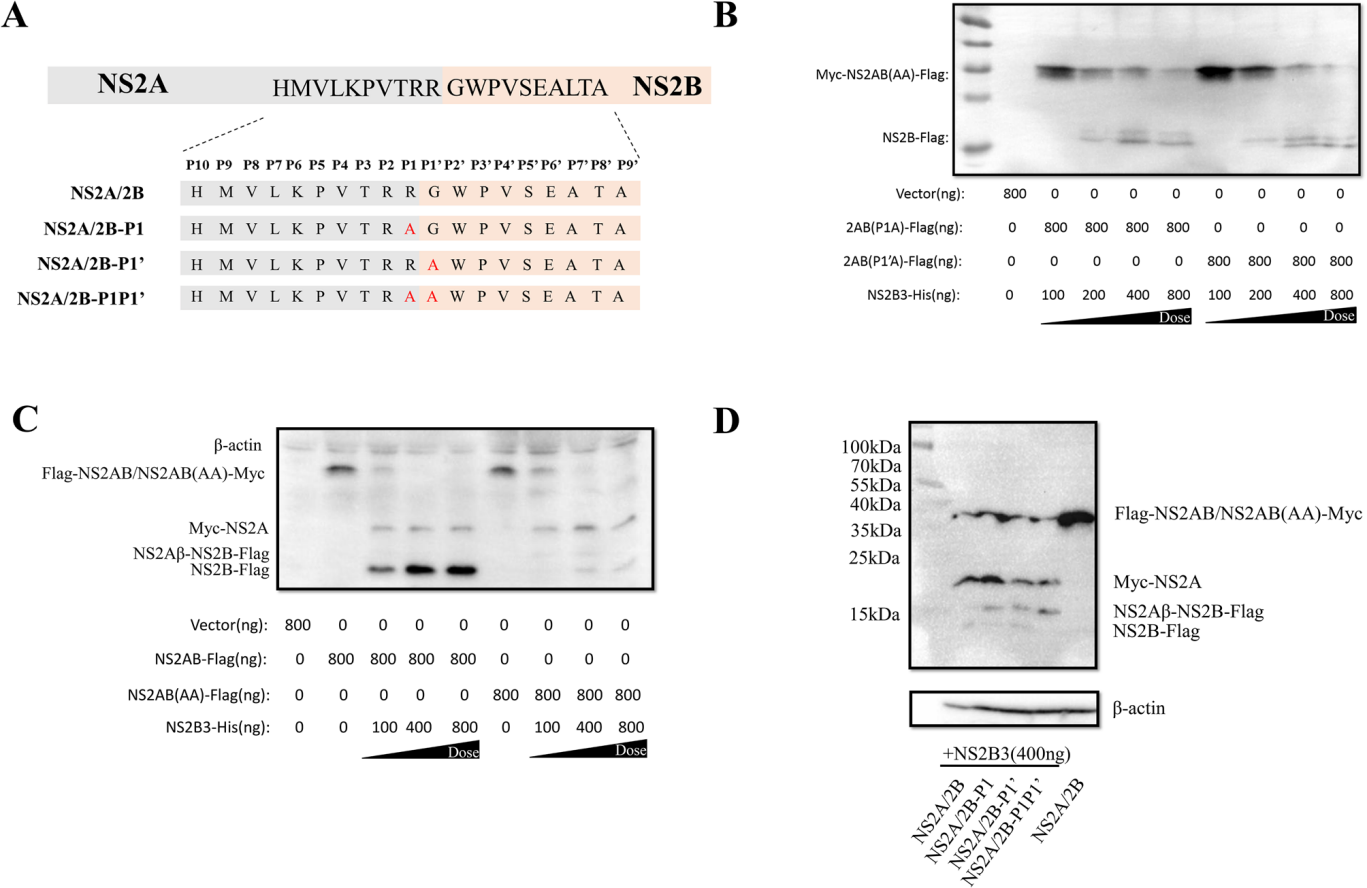
The P1 and P1′ amino acid sites of NS2A/2B are vital for NS2B3 proteolytic processing. (**A**) Schematic of plasmid construction whereby P1 and/or P1′ were mutated to Alain NS2A/2B-P. (B, C) DEFs were cotransfected with empty vector or a cleavage construct (NS2A/2B-P1, NS2A/2B-P1′ or NS2A/2B-P1P1′) and different concentrations of NS2B3, and the cells were harvested 24 h post transfection for WB analysis. Mouse anti-Flag monoclonal antibody and mouse anti-Myc monoclonal antibodies were used as the primary antibodies simultaneously. (**D**) NS2A/2B, NS2A/2B-P1, NS2A/2B-P1′ or NS2A/2B-P1P1′ was cotransfected with 400 ng NS2B3 respectively and the cells were harvested 24 h post transfection for WB, Mouse anti-Flag monoclonal antibody and mouse anti-Myc monoclonal antibodies were used as the primary antibodies simultaneously.

To determine whether P1 and P1′ amino acids are needed for its cleavage, the P1 and/or P1′ amino acid sites of DTMUV NS2A/2Bw ere mutated into Ala as shown in Fig. 4A. When DEFs were cotransfected with pCAGGS-Myc-NS2A/2B-Flag and 400 ng of pCAGGS-NS2B3-His, NS2A/2B could be completely cleaved into NS2A and NS2B. However, when DEFs were cotransfected with pCAGGS-Myc-NS2A/2B(P1A)-Flag or pCAGGS-Myc-NS2A/2B(P1′A)-Flag and 400 ng of pCAGGS-NS2B3-His, neither NS2A/2B(P1A) nor NS2A/2B(P1′A) could be completely cleaved by NS2B3, and the cleavage efficiency was largely decreased, even at high concentrations(Fig. 4B). To our surprise, when both the P1 and P1′ sites were mutated to Ala, their influence on NS2A/2B cleavage was much weaker than the single mutation at P1 or P1′ (Fig. 4C). Then, 400 ng of pCAGGS-NS2B3-His was cotransfected with pCAGGS-Myc-NS2A/2B-Flag, pCAGGS-Myc-NS2A/2B(P1A)-Flag, pCAGGS-Myc-NS2A/2B(P1′A)-Flag or pCAGGS-Myc-NS2A/2B(P1P1′AA)-Flag plasmids and find that all the mutation can attenuate the cleavage between NS2A and NS2B(Fig. 4D).

### The P1 and P1′ amino acid sites of NS2A/2B strongly affect DTMUV replication

**Figure 5:**
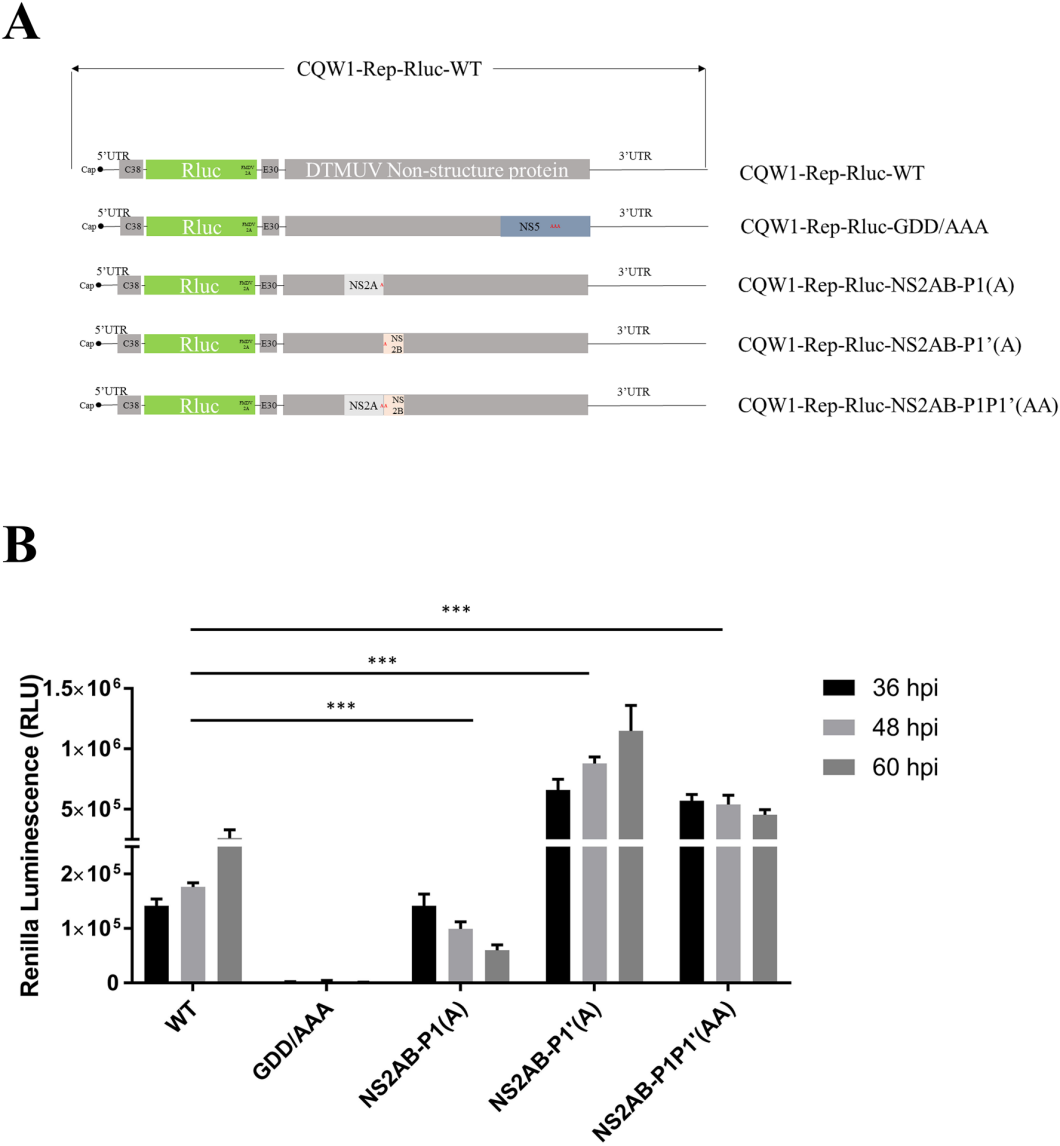
The P1 and P1′ amino acid sites of NS2A/2B strongly affect DTMUV replication. (**A**) Schematic of the construction of DTMUV mutation replicons. (**B**) BHK21 cells were transfected with the replicons and harvested at 36, 48 and 60 h post transfection. Then, the replication of DTMUVmut replicons was evaluated by measuring the Renillaluciferase level, CQW1-Rep-Rluc-GDD/AAA was used as negative control. Significant differences from the mock groups are indicated by *P < 0.05, **P < 0.01 and ***P < 0.001.

To verifying whether the P1 and P1′ sites are important to the viral replication, DTMUV replicons with mutated P1 and/or P1′ sites were constructed (Fig. 5A). BHK21 cells were transfected with CQW1-Rep-Rluc-WT, CQW1-Rep-Rluc-GDD/AAA(the active sites of RNA-dependent RNA polymerase were mutated to A which cause lethal influence on replicon and used as negative control), CQW1-Rep-Rluc-NS2A/2B-P1(A), CQW1-Rep-Rluc-NS2A/2B-P1′(A), or CQW1-Rep-Rluc-NS2A/2B-P1P1′(AA) and the cells were harvested at 36 h, 48 h, and 60 h post transfection. Finally, the RLuc expression from the replicons was measured. As shown in Fig. 5B, when Arg at the P1 site was substituted by Ala, there plication of the DTMUV mutated replicon was largely blocked. When both the P1 and P1′ sites were substituted by Ala, there plication of the DTMUV mutated replicon as represented by luciferase activity showed a high Rluc level at early times post transfection, but it did not continue to increase. However, it is interesting to mention that the replication of DTMUV-P1′(A) replicon was even better than that of the DTMUV-WT replicon.

### The P1 and P1′ amino acid sites of NS2A/2B are important for DTMUV proliferation and virulence

**Figure 6:**
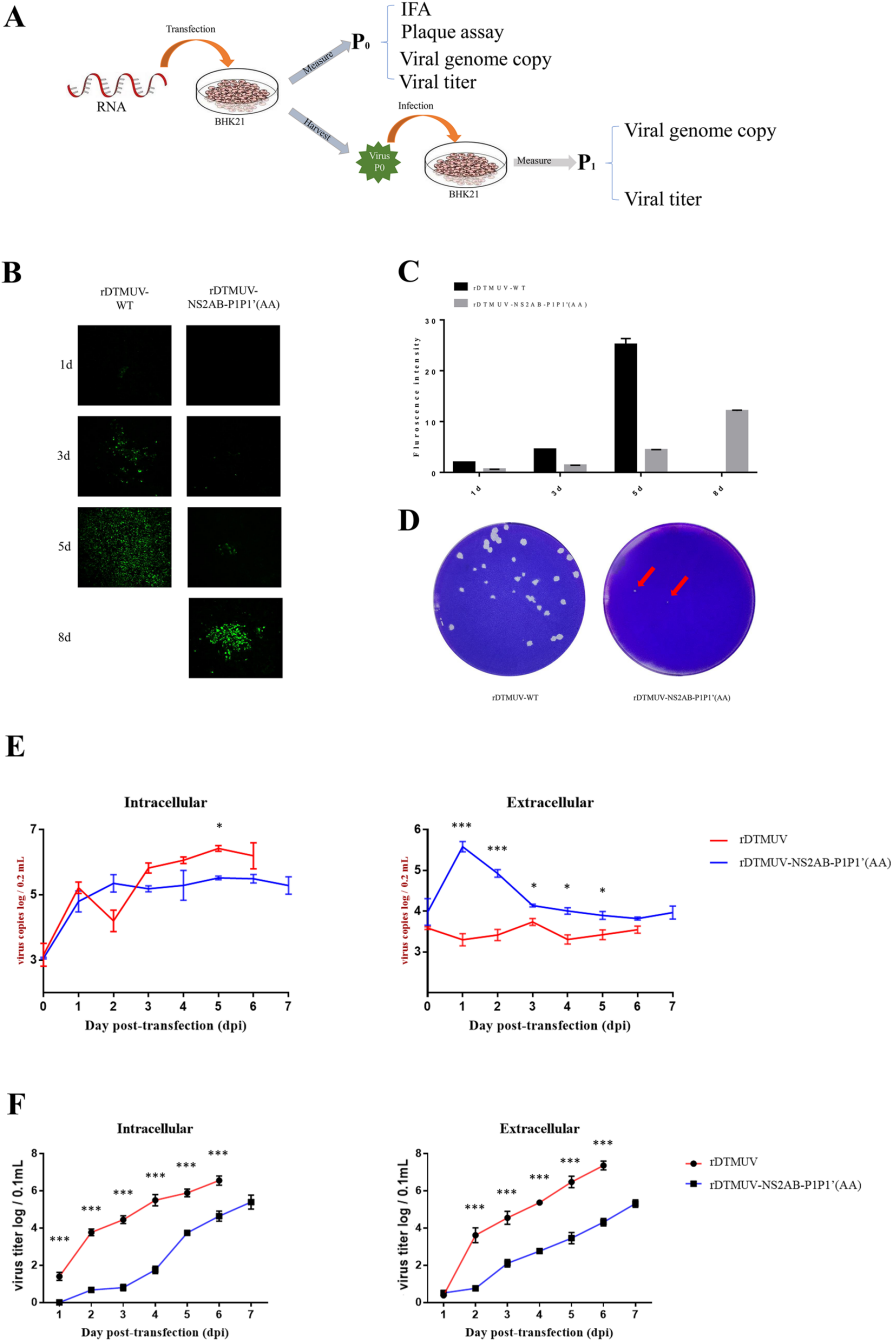
The P1 and P1′amino acidsites of NS2A/2B are important for P0 DTMUV proliferation. (**A**) Schematic of the recombinant viruses rescue and data collection. (**B)** IFA detection of rDTMUV-WT and rDTMUV-NS2A/2B-P1P1′(AA). BHK21 cells were transfected with rDTMUV RNA, and the cells were harvested 1, 3, 5, 8 days post transfection for IFA, and mouse anti-DMTUV polyclonal antibody was used as the primary antibody. (**C**) Quantitative annals of IFA data in Fig B. (**D**) Plaque assay for rDTMUV-WT and rDTMUV-NS2A/2B-P1P1′(AA). rDTMUV-WT caused larger plaque sizes than rDTMUV-NS2A/2B-P1P1′(AA) at 4 days post transfection. (**E**) Genome copy numbers of both intracellular and extracellular rDTMUV-WT and rDTMUV-NS2AB-P1P1′(AA) samples. (**F**) Growth curves of both intracellular and extracellular rDTMUV-WT and rDTMUV-NS2AB-P1P1′(AA) samples. The viral genome copy number and TCID_50_ of each sample were measured at different time points. All data are represented as the mean ± SEM (n = 4). Significant differences from the mock groups are indicated by *P < 0.05, **P < 0.01 and ***P < 0.001.

**Figure 7:**
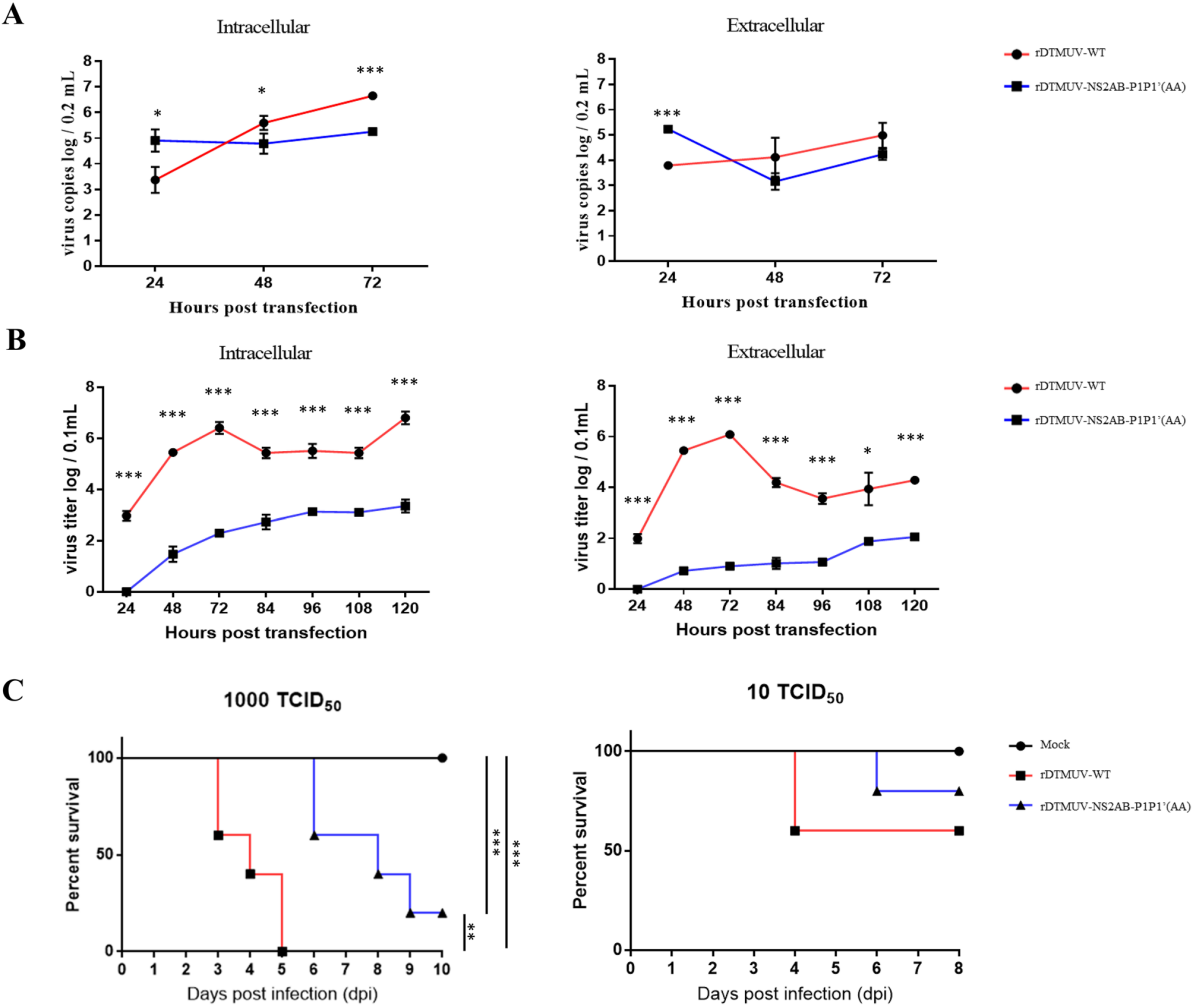
The P1 and P1′amino acid sites of NS2A/2B are important for P1 DTMUV proliferation and virulence. BHK21 cells were infected with P0 rDTMUV-WT and P0 rTMUVrDTMUV-NS2A/2B-P1P1′(AA) at an infectious dose of 100 TCID_50_ in 12-well plates, and viral titers were measured at each time point using the TCID_50_ message on BHK21 cells. (**A**) Genome copy number of the intracellular and extracellular sample of cells infected with two recombination viruses. (**B**) The viral titers of the intracellular and extracellular sample of cells infected with two recombinant viruses. (**C**) Virulence of rDTMUV-WT and rTMUVrDTMUV-NS2A/2B-P1P1′(AA) in 9-day-old duck embryonated eggs. All data are represented as the mean ± SEM (n = 4). Significant differences from the mock groups are indicated by *P < 0.05, **P < 0.01 and ***P < 0.001.

To verifying whether the P1 and P1′ sites are important to the viral proliferation and virulence, infection clone was used in experiment. A schematic of the experimental setup and data collection is shown in Fig. 6A. Based on our replicon data, we found that the replication of CQW1-Rep-Rluc-NS2A/2B-P1P1′(AA) was not inhibited by the mutations and was even better than that of CQW1-Rep-Rluc-WT 36 h post transfection (Fig. 5B). Therefore, we wanted to determine the effects of the mutations on the virulence of a recombinant virus. P1 and P1′ site mutations were introduced into an infectious recombinant DTMUV, which was rescued from BHK21 cells and was named rDTMUV-NS2AB-P1P1′(AA). Interestingly, the CPE and virus infection of transfected BHK21 were delayed as observed by indirect immunofluorescence using a mouse anti-DMTUV polyclonal antibody as the primary antibody (Fig. 6B, C). Plaque assay (Fig. 6D) demonstrated that rDTMUV-WT caused clear and distinct plaques at 72 h post infection, while rDTMUV-NS2AB-P1P1′(AA) can only cause two smaller plaques at the same time point in cells infected with the same TCID_50_. Moreover, the plaque size of rDTMUV-NS2A/2B-P1P1′(AA) was smaller than that of rDTMUV-WT. Then, growth curves and genome copy numbers of both intracellular and extracellular rDTMUV-WT and rDTMUV-NS2AB-P1P1′(AA) were measured, as shown in Fig. 6E and F. We found that the copy number of rDTMUV-NS2A/2B-P1P1′(AA) was only slightly lower than that of rDTMUV-WT; however, the viral titer of rDTMUV-NS2A/2B-P1P1′(AA) was much lower than that of rDTMUV-WT. To better understand this discrepancy, we performed one more round of virus infection, and infected BHK21 cells with rDTMUV-NS2A/2B-P1P1′(AA). The same trends in growth curve and genome copy number were observed (Fig. 7A, B).

### Virulence of recombination virus in duck embryos

The virulence of rDTMUV-WT and rDTMUV-NS2A/2B-P1P1′(AA) was evaluated in 9-day-old duck embryos. When duck embryos were infected with 1,000 TCID_50_, all rDTMUV-WT-infected duck embryos died within 5 days, while no rDTMUV-NS2A/2B-P1P1′(AA)-infected duck embryos died within 5 days (Fig. 7C). However, 80% rDTMUV-NS2A/2B-P1P1′(AA)-infected duck embryos died within 10 days (Fig. 7C). In an experiment with a lower virus challenge (10 TCID_50_), 40% rDTMUV-WT infected duck embryos died within 8 days, while 20% rDTMUV-NS2A/2B-P1P1′(AA)-infected duck embryos died within 8 days (Fig. 7C), there was significantly difference of two group by statistical analysis.

## Discussion

NS2A and NS2B participate in the construction of the replication complex and play an essential role in virus replication. NS2A is a membrane protein with 8 transmembrane domains located on the ER that can assist in the reconstruction of the ER^18^. NS2B is also a transmembrane protein, and the junction between NS2B and NS3 is self-cleaved by the viral protease, whereby NS2B binding to NS3 causes conformational changes in NS3, which converts NS3 into the more active and stable NS2B3 proteinase^19,20^. It has been reported that flavivirus NS2B3pro cleaves the viral NS2A/2B, but the key element of the proteolytic process between NS2A and NS2B has barely been discussed. Duck Tembusu virus is a newly emerging flavivirus. In this study, we mutated the NS2A/2B P1 and P1′ sites to alanine and the proteolytic process was decrease by this substitute, the products of the proteolysis of NS2A/2B(P1P1′-AA) seems less than other groups, which suggested that the double mutation can weak proteolytic process, but it isn’t be abolished. But there are two sites can be processed by NS2B3pro, NS2Aα could been so formed. Accordingly, cleavage of NS2A/2B results in NS2Aα, NS2Aβ, NS2A(due to inner incomplete cleavage) and NS2B protein. However, NS2Aβ can’t be detected because it bears no tag. Moreover, NS2A/2B(P1P1′-AA) mutant cleavages into NS2Aα, NS2Aβ, NS2A, NS2Aβ-2B (due to incomplete cleavage), and NS2B protein. So, cleavage of NS2A/2B and NS2A/2B(P1P1′-AA) mutant do not result in the equivalent amount of cleavage proteins in WB picture. We confirmed that P1 (R) and P1′ (G) of DTMUV NS2A/2B are vital for the proteolytic processing by NS2B3. Moreover, the present of three amino acid (P2 (R), P3 (T), and P4 (V)) is also important for this proteolytic process. However, P5 (P), P6 (K), P7 (L), P8 (V), P9 (M), and P10 (H) exert mild influence on this process. According to this result, we conclude that the amino acids closer to the NS2A/2B cleavage site showed greater effects on its cleavage.

In other reports, two fluorophores were linked with the cleaved amino acids to detect cleavage efficiency; however, the studies did not show whether the first protein or the second protein is needed *in cis* for its cleavage^21,22^. In most studies, the efficiency of the proteolytic process has been detected by using different extrinsic fluorophores to mimic NS polyprotein cleavage by NS2B3; however, this assay does not explain the real relationship between the substrate NS protein and the protease NS2B3. In our study, NS2A or NS2B were substituted with EGFP or GST protein, respectively, forming different polyprotein combinations such as EGFP-20aa-GST, EGFP-20aa-NS2B, and NS2A-20aa-GST. We found that EGFP-20aa-GST and EGFP-20aa-NS2B could not be cleaved by NS2B3, while NS2A-20aa-NS2B and NS2A-20aa-GST could. Further study confirmed that NS2A could be intermolecularly cleaved by NS2B3 regardless of whether NS2A was linked with NS2B or GST. Collectively, these results demonstrate that both NS2A and NS2B are *in cis* needed for NS2A/2B intramolecular protease cleavage, while NS2A could be intermolecularly processed by NS2B3.

It has been reported that NS2A can be intermolecularly cleaved by NS2B3 of YFV and DENV to form NS2Aα^14,23–25^. This is similar to what we found here that DTMUV NS2A could be intermolecularly processed by NS2B3 to form NS2Aα and NS2Aβ, and NS2Aα/β could be cleaved by NS2B3pro in trans. According to previous reports, this cleavage site is important for YFV virus particle assembly and the production of infectious particles^14^. In the flavivirus life cycle, NS2A/2B cleavage produces NS2A, and NS2A first plays a role in viral genome replication. Then, NS2A is cleaved into NS2Aα, which is important for progeny virus assembly. Therefore, the NS2Aα/β cleavage should take place after NS2A/2B cleavage. However, in our study, we found that NS2Aα/β could be cleaved by NS2B3 regardless of a prior NS2A/2B cleavage. This result indicates that NS2Aα/β cleavage is independent of NS2A/2B cleavage the NS2Aα/β cleavage can occurs before NS2A/2B if the NS2A/2B cleavage site was modified. By comparing the amino acid sequence of DTMUV and the reported sequence of YFV, we speculate that the NS2Aα/β cleavage site in DMTUV is KK_194_↓G_195_, two basic amino acids and a short chain amino acid; however, the experimentally confirmed sites of NS2Aα/β in DTMUV still need to be verified. Additionally, the function of NS2Aα in the DTMUV life cycle should be studied.

The P1 to P10 sites and the P1′ site alanine mutations showed different effects on NS2A/2B cleavage. To understand their effects on viral proliferation, we chose to mutate the P1 and/or P1′ sites (RG/AG, RG/RA and RG/AA) of a DTMUV replicon to best maintainNS2A/2B function and structure. We found that when the production of mature NS2A and NS2B decreased, viral genome replication decreased. Interestingly, our data showed that CQW1-Rep-Rluc-NS2A/2B-P1′(A) replicated better than CQW1-Rep-Rluc-NS2A/2B-WT, while the production of NS2A and NS2B decreased when P1′was mutated to Ala. Therefore, we hypothesize that the P1′ site of NS2A/2B possesses unknown functions that can improve viral RNA replication.

Because P1 mutations had a detrimental effect on the replicon, we chose to mutate the P1 and P1′ sites of a DTMUV infectious clone. Consistent with previous results, rDTMUV-NS2AB-P1P1′(AA) was attenuated both in vitro and in vivo, showing lower viral genome copies and titers, as well as a lower mortality in duck embryos. In the IFA assay, we found that rDTMUV-NS2AB-P1P1′(AA) could only infect a portion of the cells throughout the experiment, which suggested that the mutation affected their plication and any other step of viral life cycle. However, we do not know the exact mechanism by which this mutation affects virus attachment, assembly or release.

In summary, by mutating the cleavage sites, we found that 5 amino acids (P1-R, P1′-G, P2-R, P3-T, and P4-V) are vital for NS2A/2B cleavage. In addition, both NS2A and NS2B were essential *in cis* for polyprotein NS2A/2B intramolecular cleavage, while NS2Aα intermolecular proteinase cleavage was NS2B independent. Subsequently, our data on a DTMUV replicon and an infectious clone showed that the P1 site is essential to DTMUV genome RNA replication, while mutation of P1′ could boost viral replication. Furthermore, a recombinant virus with P1 and P1′ site mutations, named rDTMUV-NS2A/2B-P1P1′(AA), was rescued from transfected BHK21 cells. rDTMUV-NS2A/2B-P1P1′(AA) showed CPE from 5 days and continued to increase up to 8 days post transfection, while rDTMUV-WT showed CPE from 3 days and peak at 5 days post transfection. The maximum viral titers and viral genome copies of rDTMUV-NS2A/2B-P1P1′(AA) were much lower than those of rDTMUV-WT both in the intracellular and extracellular samples of infected BHK21. The virulence of rDTMUV-NS2A/2B-P1P1′(AA) was attenuated in 9-day-old duck embryonated eggs. Taken together, the above data indicated that the mutation of the intramolecular protease cleavage sites between NA2A/2B attenuates DTMUV both in BHK21 and in duck embryonated eggs. The NS2A/2B cleavage sites processed by the NS2B3 protease are vital for DTMUV proliferation and virulence.

## Methods and material

### Cells and virus

Duck embryo fibroblasts (DEFs) and BHK21 cells were maintained in high-glucose Dulbecco’s modified Eagle’s medium (DMEM) (Gibco, Grand Island, USA) supplemented with 10% newborn calf serum (NBS) (Gibco, Grand Island, USA), 100 U/ml penicillin and 100 μg/ml streptomycin at 37 °C in 5% CO_2_. The clinical TMUV strain CQW1 (GenBank: KM233707.1) was isolated from ill Cherry Valley ducks^26^.

### Plasmid construction

Standard molecular biology methods were used in the construction of all plasmids. DTMUV RNA was extracted from infected DEFs, and then full-length cDNA of DTMUV was generated according to the manufacturer's instructions (Invitrogen). The NS2A/2B fragment was amplified by polymerase chain reaction (PCR) with the primer pair pCAGGS-Myc-NS2A/2B-F and pCAGGS-NS2A/2B-Flag-R (Table 1) from the cDNA. The NS2B3 fragment was amplified as NS2A/2B with the primer pair pCAGGS-NS2B3-F and pCAGGS-NS2B3-R-His (Table 1). A mammalian expression vector, pCAGGS, was used in this study to express fusion proteins. Restriction enzyme sites (*EcoRI*, *BilII*) of pCAGGS were used for cloning different fragments, and Myc-NS2A and GST-Flag were constructed by using the primers pair pCAGGS-Myc-NS2A/2B-F, pCAGGS-NS2A-R and pCAGGS-GST-F, pCAGGS-Flag-GST-R, respectively. The mutant plasmids were generated by a Fast Mutagenesis System (TransGen Biotech) with the primer pairs NS2A/2B-P1A-F and NS2A/2B-P1A-R, NS2A/2B-P1′A-F and NS2A/2B-P1′-R, NS2A/2B-P1AP1′A-F and NS2A/2B-P1AP1′A-R (Table 1). All NS2A/2B proteins contained a Myc tag in the N terminus and a Flag tag in the C terminus. Overlap PCR was used to construct the multisite mutation plasmids NS2A/2B-M1, and two fragments were amplified by Myc-NS2A/2B-F, NS2A/2B-M1-R and NS2A/2B-M1-F, NS2A/2B-Flag-R, respectively. Then, the two fragments were combined by PCR and assembled into pCAGGS. NS2A/2B-M2 and NS2A/2B-M3 were constructed in the same way (Fig. 3A). Finally, NS2A or NS2B was substituted by EGFP or GST, and the two proteins were linked by the 20 amino acids between NS2A and NS2B (Fig. 2A). EGFP-20aa and NS2B-Flag were amplified and combined by PCR, and EGFP-20aa-NS2B was assembled into pCAGGS. The other two proteins were constructed the same as pCAGGS-EGFP-20aa-NS2B-Flag. NS2A/2B-M1, NS2A/2B-M2 and NS2A/2B-M3 were constructed by using overlap PCR, and the primers used in this experiment are shown in Table 1.

**Table 1:** Sequences of DNA primers used in this study.

Primer name	Sequence (5′ to 3′)
pCAGGS-Myc-NS2A/2B-F	CATCATTTTGGCAAAGAATTCGCCACCATGGAGCAGAAACTCATCTCTGAAGAGGATCTGTTTCAAGGGGGTGGCATGG
pCAGGS-Myc-NS2A/2B-R	TTGGCAGAGGGAAAAAGATCTCTACTTATCGTCGTCATCCTTGTAATCTCGTTGTTTTGCCTTAGT
pCAGGS-EGFP-F	CATCATTTTGGCAAAGAATTCGCCACCATGGAGCAGAAACTCATCTCTGAAGAGGATCTGATGGTGAGCAAGGGCGAGGAG
pCAGGS-GST-Flag-R	TTGGCAGAGGGAAAAAGATCTCTACTTATCGTCGTCATCCTTGTAATCTTTTGGAGGATGGTCGCCACC
pCAGGS-GST-F	CATCATTTTGGCAAAGAATTCGCCACCATGGAGCAGAAACTCATCTCTGAAGAGGATCTGTCCCCTATACTAGGTTATTGG
pCAGGS-NS2A-R	TTGGCAGAGGGAAAAAGATCTCTACTTATCGTCGTCATCCTTGTAATCTCTCCGTGTCACTGGCTTCAG
pCAGGS-NS2B3-F	CATCATTTTGGCAAAGAATTCGCCACCATGGGGTGGCCAGTCAGTGAGGCT
pCAGGS-NS2B3-His-R	TTGGCAGAGGGAAAAAGATCTCTAATGGTGATGGTGATGATGTCTCTTTCCACTCGCAAAATC
NS2A-P1-F	GCCAGTGACACGGGCAGGGTGGCCAGTCA
NS2A-P1-R	GCCCGTGTCACTGGCTTCAGCACCATGTG
NS2A-P1′-F	GCCAGTGACACGGAGAGCGTGGCCAGTCA
NS2A-P1′-R	GCTCTCCGTGTCACTGGCTTCAGCACCAT
NS2A-P1P1′-F	GCCAGTGACACGGGCAGCGTGGCCAGTCA
NS2A-P1P1′-R	GCTGCCCGTGTCACTGGCTTCAGCACCAT
C-EGFP-20aa-GST-Flag-F	GACACGGAGAGGGTGGCCAGTCAGTGAGGCTTTGACTGCTTCCCCTATACTAGGTTATTGG
C-EGFP-20aa-GST-Flag-R	CTGGCCACCCTCTCCGTGTCACTGGCTTCAGCACCATGTGCTTGTACAGCTCGTCCATGCC
C-EGFP-20aa-2B-Flag-F	TGAAGCCAGTGACACGGAGAGGGTGGCCAGTCAGTGAGGCT
C-EGFP-20aa-2B-Flag-R	TCTCCGTGTCACTGGCTTCAGCACCATGTGCTTGTACAGCTCGTCCATGCC
C-Myc-NS2A-20aa-GST-Flag-F	GGGTGGCCAGTCAGTGAGGCTTTGACTGCTTCCCCTATACTAGGTTATTGG
C-Myc-NS2A-20aa-GST-Flag-R	GCCTCACTGACTGGCCACCCTCTCCGTGTCACTGGCTTCAG
C-NS2A/2B-M1-F	CTGCACATGGTGCTGAAGCCAGCGGCAGCGAGAGGG
C-NS2A/2B-M1-R	CTCACTGACTGGCCACCCTCTCGCTGCCGCTGGCTT
C-NS2A/2B-M2-F	TTTGCTGGTCTGCACATGGTGGCGGCGGCAGTGACA
C-NS2A/2B-M2-R	TGGCCACCCTCTCCGTGTCACTGCCGCCGCCACCAT
C-NS2A/2B-M3-F	CCTTTGGTGTTTGCTGGTCTGGCCGCGGCGCTGAAG
C-NS2A/2B-M3-R	TCTCCGTGTCACTGGCTTCAGCGCCGCGGCCAGACC

### Mutated replicon and infection clone construction

The mutant plasmids were produced by a Fast Mutagenesis System (TransGen Biotech, Beijing, China). The DTMUV replicon was provided by our lab. Three different mutated replicons were constructed and named CQW1-Rep-Rluc-WT-NS2A/2B-P1(A), CQW1-Rep-Rluc-WT-NS2A/2B-P1′(A), and CQW1-Rep-Rluc-WT-NS2A/2B-P1P1′(AA) (Fig. 4A). Then, the plasmids were used to transfect BHK21 cells and the cells were harvested at different times post transfection. The replication of the replicon was measured with a Renilla luciferase reporter located upstream of the replicon. The plasmid pACYC FL-TMUV containing the full-length cDNA of DMTUV was provided by our lab^27^. Then, the P1 and P1′ sites were mutated to Ala by using a Fast Mutagenesis System (TransGen Biotech, Beijing, China), and the mutated plasmid was named pACYC FL-TMUV-P1P1′(AA).

### Renilla luciferase activity assay

BHK21 cells transfected with WT or mutant replicon plasmids were lysed using cell lysis buffer (Promega, USA) and harvested at various time points. A Renillaluciferase assay system (Promega) and a Varioskan Flash luminometer (Thermo Scientific, USA) were used to detect the Rluc activity according to the manufacturer's instructions.

### In vitro RNA transcription and transfection

The plasmids pACYC FL-TMUV and pACYC FL-TMUV-P1P1′(AA) were linearized by the restriction enzymes *NotII* or *SmaII* and purified by using the TaKaRaMiniBEST DNA Fragment Purification Kit (Takara, Japan). Then, the linearized DNA was transcribed to RNA with mMESSAGE mMACHINE T7 Transcription Kit (Ambion, USA). For transfection, BHK21 cells were plated on 6-well plates, and after culture at 37 °C for 16 h, the cells were transfected with 2.0 μg RNA per well with TransIntro EL Transfection Reagent (TransGen Biotech, Beijing, China). After transfection, the cells were cultured at 37 °C with 5% CO_2_. Then, the transfected cells were observed and harvested until cytopathic effect (CPE) appeared. rDTMUV-WT and rDTMUV-NS2A/2B-P1P1′(AA) were stored for the subsequent experiments.

### Transfection of cells with plasmids

*TransIntro*™ EL Transfection Reagent (TransGen Biotech, Beijing, China) was used in this experiment. DEFs were transfected with the plasmid pCAGGS-Myc-NS2A/2B-Flag and harvested at 24, 36 and 48 h post transfection. Cleavage of NS2A/2B by NS2B3 was verified by co-transfection, whereby DEFs were cotransfected with NS2A/2B and different doses of NS2B3, and the cells were harvested after 24 h.

### Western blot assay

All cell samples were analyzed by western blot. The samples were separated by SDS-PAGE and transferred to polyvinylidene difluoride (PVDF) membranes. Following incubation in blocking buffer (5% skim milk), the membrane was washed 3 times with TBST and incubated in blocking buffer containing a mouse monoclonal antibody against Flag tag (TransGen Biotech, Beijing, China). Then, the membranes were washed 3 times with TBST and incubated with a goat anti-mouse antibody conjugated to horseradish peroxidase (TransGen Biotech, Beijing, China). Finally, the membrane was washed 3 times with TBST buffer, and the antibody-protein complexes were detected using the Clarity Western ECL Substrate (Bio-Rad).

### Indirect immunofluorescence assay (IFA)

BHK21 cells grown on cover slips were transfected with the virus genome and collected daily. After washing with PBST, the cells were fixed with 1% paraformaldehyde for 1 h, permeabilized with 0.22% Triton X-100 for 20 min at 4 °C, and blocked with 5% BSA. Then, mouse anti-TMUV polyclone was used as the primary antibody at a dilution of 1:200 with 1% BSA. Cells were incubated at 37 °C for 2 h, washed with PBS, and incubated with fluorescein isothiocyanate (FITC)-labeled goat anti-mouse IgG at a dilution of 1:200 for 1 h. Finally, the cells were analyzed under a fluorescence microscope (Nikon, Tokyo, Japan).

### Virus titration and plaque assay

Virus titers were measured by the median tissue culture infectious dose 50 (TCID_50_) method on BHK21 cells. The virus was serially diluted tenfold in DMEM, and each dilution was distributed to 8 wells of a 96-well plate. Then, the culture was incubated at 37 °C with 5% CO_2_ for 7 days. Next, the cells were detected by microscopy, and the viral titers were calculated according to the Karber method. For the plaque assay, BHK21 cells were cultured in DMEM in a 6-well plate for 16 h, and then the medium was removed and the cells were washed with PBS three times. Virus samples were diluted and added to the cells with the same TCID_50,_ and then the samples were incubated for 1.5 h and swirled every 15 min. After the incubation, the virus sample was removed, and the cells were washed with PBS 3 times. Finally, 2 ml of 0.75% methyl cellulose overlay containing 2% FBS and 1% penicillin/streptomycin was added to each well. At 3 days post infection, the medium was removed, and the cells were washed 3 times with PBS and fixed with 4% paraformaldehyde for 20 min. After the fixative was removed and the cells were washed with PBS, the cells were stained with 1% crystal violet and washed carefully. Then, plaques could be observed in the plate.

### Virulence of rDTMUV in duck embryos

All duck embryos were purchased from the Waterfowl Breeding Center of Sichuan Agriculture University and randomly divided into 3 groups. Five 9-day-old embryo eggs per group were infected with a 100-μL dilution of rDTMUV-WT or rDTMUV-NS2A/2B-P1P1′(AA) by allantoic cavity inoculation with 1,000 or 10 TCID_50_, and DMEM was used as a negative control. Then, the embryos were incubated at 37 °C for 10 days and checked daily with an egg candler. If the embryos had stopped moving and did not have clear blood vessels, the embryos were regarded as dead.

### Ethics statement

This study was approved by the Committee of Experiment Operational Guidelines and Animal Welfare of Sichuan Agricultural University. Experiments were conducted in accordance with approved guidelines.

## Supplementary information

Supplementary figures.
