# Author Correction: Determinants of duck Tembusu virus NS2A/2B polyprotein procession attenuated viral replication and proliferation in vitro

**DOI:** 10.1038/s41598-021-03536-w

**Published:** 2021-12-16

**Authors:** Bowen Jiang, Wei Zhang, Yuanyuan Wu, Tao Wang, Mingshu Wang, Renyong Jia, Dekang Zhu, Mafeng Liu, Xinxin Zhao, Qiao Yang, Ying Wu, ShaQiu Zhang, YunYa Liu, Ling Zhang, YanLing Yu, Leichang Pan, Shun Chen, Anchun Cheng

**Affiliations:** 1 Research Center of Avian Disease, College of Veterinary Medicine, Sichuan Agricultural University, Chengdu, 611130 Sichuan China; 2 Institute of Preventive Veterinary Medicine, Sichuan Agricultural University, Chengdu, 611130 Sichuan China; 3 Key Laboratory of Animal Disease and Human Health of Sichuan Province, Chengdu, 611130 Sichuan China

Correction to: *Scientifc Reports* 10.1038/s41598-020-68271-0, published online 24 July 2020

The original version of this Article contained an error in Figure 1D where the label indicating “NS2B-Flag(14kDa)” was incorrectly given as “Myc-NS2B(25kDa).”

The original Figure 1 and accompanying legend appear below.

**Figure 1:**
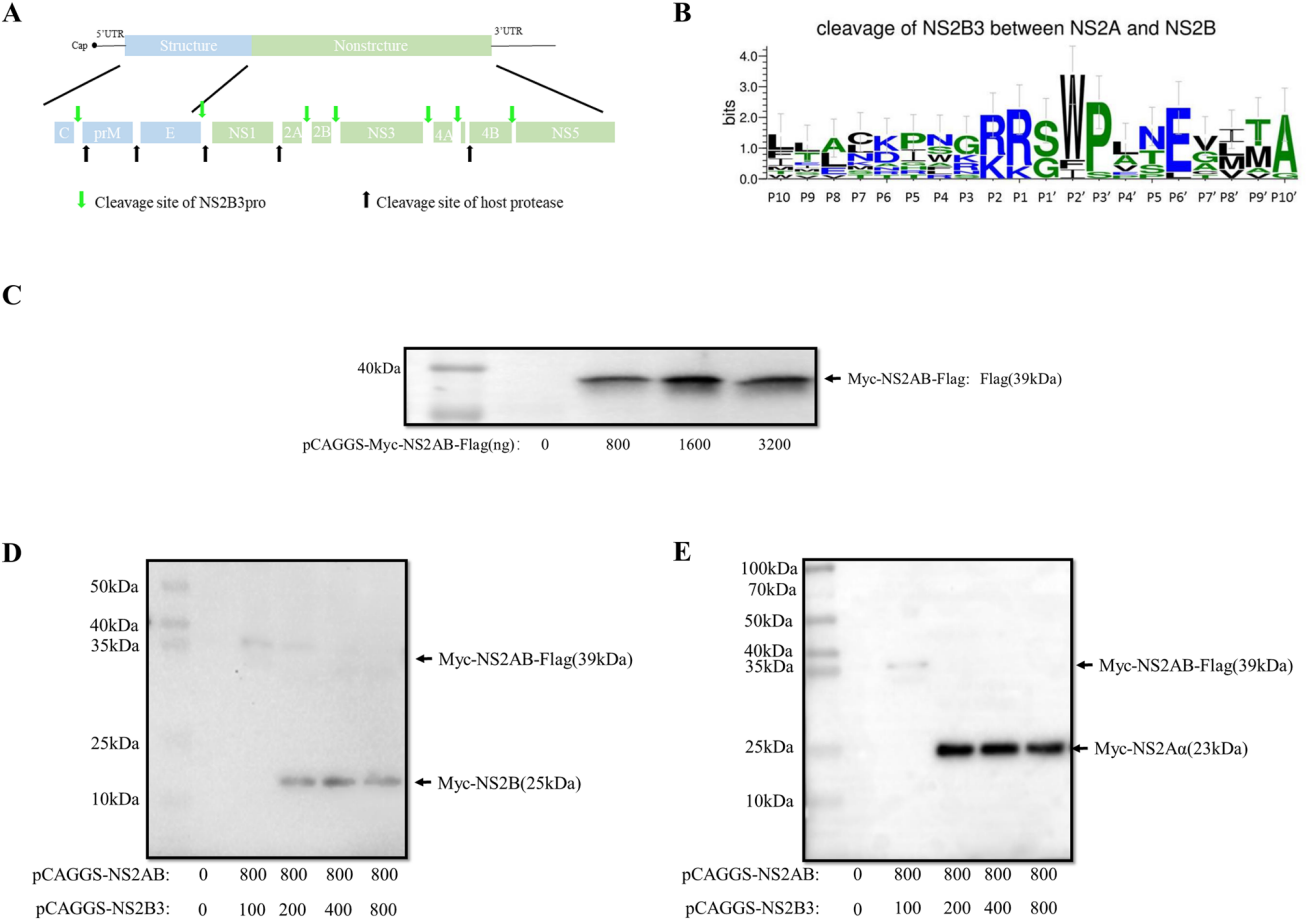
Cleavage of NS2A/2B by NS2B3. (**A**) Genome structure and cleavage sites of flavivirus polyprotein. (**B**) Conservative amino acid residues near the NS2A/2B cleavage site by comparison of different flaviviruses, including DENV, JEV WNV, YFV, TBEV, BGAV, ZIKA and KUN. (C) Overexpression of DTMUV NS2A/2B in transfected DEFs. DEF cells were transfected with different concentrations of pCAGGS-Myc-NS2A/2B-Flag and the cells were harvested 24 h post transfection. (**D**,** E**) Cleavage of NS2A/2B by NS2B3. DEF cells were cotransfected with plasmids expressing NS2A/2B and with different concentrations of NS2B3 plasmids, and proteins of interest were detected by WB 24 h post transfection. (**D**) Mouse anti-Flag monoclonal antibody was used as the primary antibodies, (**E**) Mouse anti-Myc monoclonal antibody was used as the primary antibody.

The original Article has been corrected.

