# Supplementary material for: Determinants of duck Tembusu virus NS2A/2B polyprotein procession attenuated viral replication and proliferation in vitro
Source: Sci Rep. 2020 Jul 24;10:12423. doi: 10.1038/s41598-020-68271-0 (PMC7381675; doi:10.1038/s41598-020-68271-0)
Supplement: Supplementary file 1 — Supplementary figures. [file 41598_2020_68271_MOESM1_ESM.docx]

**Determinants of duck Tembusu virus NS2A/2B polyprotein procession attenuated viral replication and proliferation in vitro**

Bowen Jiang^1¶^, Wei Zhang^1¶^, Yuanyuan Wu^1^, Tao Wang^1^, Mingshu Wang^1,2,3^, Renyong Jia^1,2,3^, Dekang Zhu^2,3^, Mafeng Liu^1,2,3^, Xinxin Zhao^1,2,3^, Qiao Yang^1,2,3^, Ying Wu^1,2,3^, ShaQiu Zhang^1,2,3^, YunYa Liu^1^, Ling Zhang^1^, YanLing Yu^1^, Leichang Pan^1^, Shun Chen^1,2,3,*^ , Anchun Cheng^1,2,3,*^

^1^Research Center of Avian Disease, College of Veterinary Medicine, Sichuan Agricultural University, Chengdu, Sichuan, 611130, China;

^2^Institute of Preventive Veterinary Medicine, Sichuan Agricultural University, Chengdu, Sichuan, 611130, China;

^3^Key Laboratory of Animal Disease and Human Health of Sichuan Province, Chengdu, Sichuan, 611130, China

^¶^These authors contributed equally to this work as first authors.

*Corresponding authors.

.E-mail: shunchen@sicau.edu.cn; Anchun Cheng: chenganchun@vip.163.com


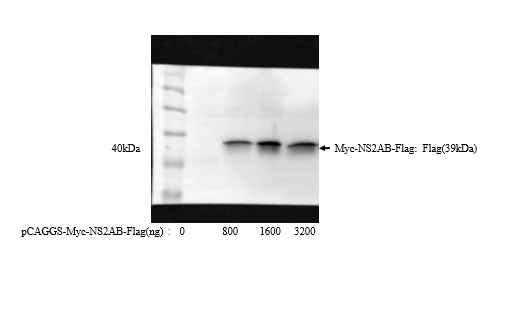


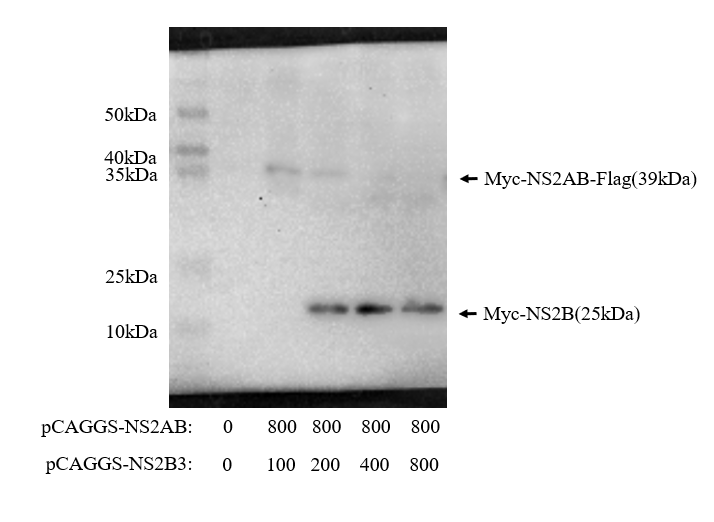


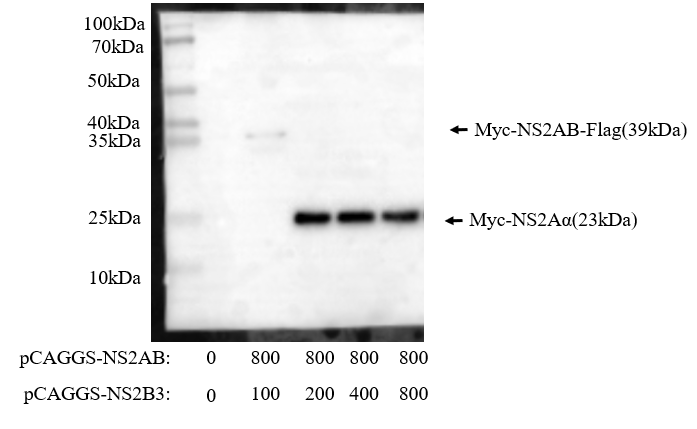
Fig. 1. Cleavage of NS2A/2B by NS2B3. (A) Genome structure and cleavage sites of flavivirus polyprotein. (B) Conservative amino acid residues near the NS2A/2B cleavage site by comparison of different flaviviruses, including DENV, JEV WNV, YFV, TBEV, BGAV, ZIKA and KUN. (C) Overexpression of DTMUV NS2A/2B in transfected DEFs. DEF cells were transfected with different concentrations of pCAGGS-Myc-NS2A/2B-Flag and the cells were harvested 24 h post transfection. (D,E) Cleavage of NS2A/2B by NS2B3. DEF cells were cotransfected with plasmids expressing NS2A/2B and with different concentrations of NS2B3 plasmids, and proteins of interest were detected by WB 24 h post transfection. Mouse anti-Flag monoclonal antibody and Mouse anti-Myc monoclonal antibody were used as the primary antibodies simultaneously


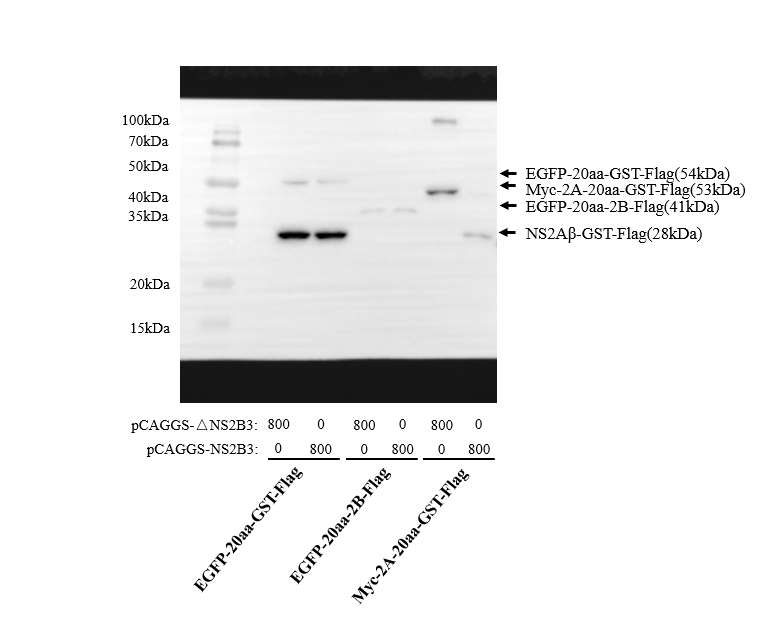


NS2A: 25kDa NS2B: 14kDa EGFP: 25kDa GST: 26kDa EGFP-20aa-GST: 50kDa 2A-20aa-GST: 49 kDa EGFP-20aa-2B:39 kDa NS2Aβ-GST: 26kDa


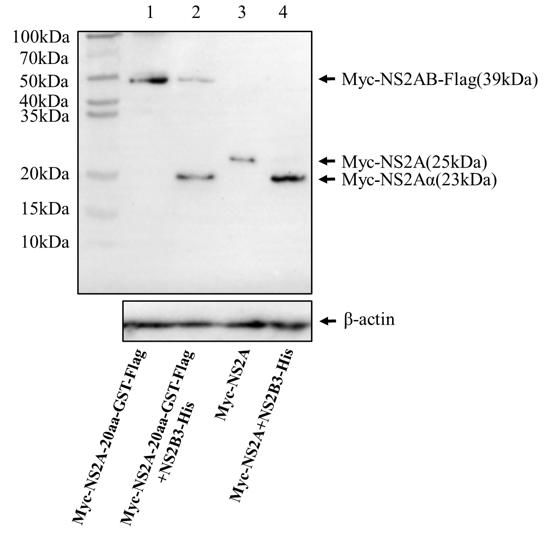


NS2A: 25kDa NS2B: 14kDa EGFP: 25kDa GST: 26kDa EGFP-20aa-GST: 50kDa 2A-20aa-

GST: 49 kDa EGFP-20aa-2B:39 kDa NS2Aβ-GST: 26kDa


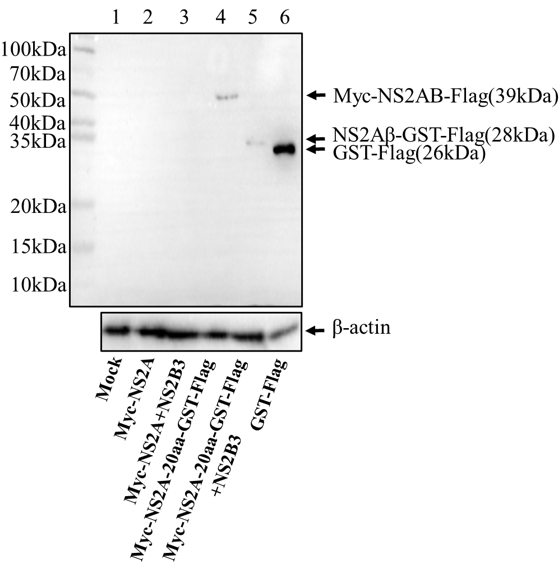


NS2A: 25kDa NS2B: 14kDa EGFP: 25kDa GST: 26kDa EGFP-20aa-GST: 50kDa 2A-20aa-

GST: 49 kDa EGFP-20aa-2B:39 kDa NS2Aβ-GST: 26kDa

Fig. 2. Both NS2A and NS2B are *in cis* needed for NS2A/2B cleavage, while DTMUV NS2B3pro can cleave NS2A. (A) Schematic of three different constructs. NS2A or NS2B were replaced by EGFP or GST, respectively, and the two were linked by 20 amino acids. (B) The constructs were coexpressed with NS2B3 in DEFs and harvested 24 h post transfection for WB analysis. Mouse anti-Flag monoclonal antibody was used as the primary antibody. Line 1: mock cells transfected with empty vector; line 2: cells transfected with EGFP-20aa-GST and empty vector; line 3: cells transfected with EGFP-20aa-GST and NS2B3; line 4: cells transfected with 2A-20aa-GST and empty vector; line 5: cells transfected with 2A-20aa-GST and NS2B3; line 6: cells transfected with EGFP-20aa-2B and empty vector; line 7: cells transfected with EGFP-20aa-2B and NS2B3; line 8: cells transfected with NS2A/2B and empty vector; line 9: cells transfected with NS2A/2B and NS2B3. (C) DEFs were transfected with different construct plasmids and harvested 24 h post transfection for WB analysis. Mouse anti-Flag monoclonal antibody and mouse anti-Myc monoclonal antibody were used as the primary antibodies simultaneously. Line 1: DEF cells were transfected with NS2A; line 2: DEF cells were transfected with NS2A and NS2B3; line 3: DEF cells were transfected with NS2A-20aa-GST; line 4: DEF cells were transfected with NS2A-20aa-GST and NS2B3. (D) DEFs were transfected with different construct plasmids and harvested 24 h post transfection for WB analysis. Mouse anti-Flag monoclonal antibody was used as the primary antibody. The GST plasmid was used as a positive control to evaluate the molecular weight of the cleaved proteins (NS2Aβ-GST). Line 1: mock cells were transfected with empty vector; Line 2: DEF cells were transfected with NS2A; line 3: DEF cells were transfected with NS2A and NS2B3; line 5: DEF cells were transfected with GST; line 5: DEF cells were transfected with NS2A-20aa-GST; and line 6: DEF cells were transfected with NS2A-20aa-GST and NS2B3.


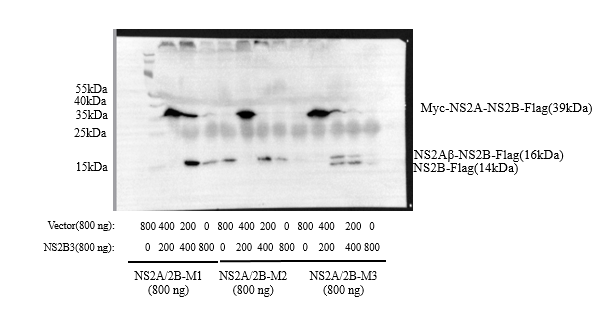


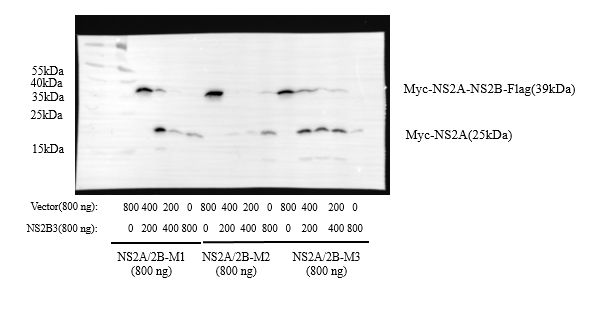


NS2A: 25kDa NS2B: 14kDa EGFP: 25kDa GST: 26kDa EGFP-20aa-GST: 50kDa 2A-20aa-

GST: 49 kDa EGFP-20aa-2B:39 kDa NS2Aβ-GST: 26kDa

Fig. 3. The P2-P4 amino acid sites of DTMUV NS2A/2B differentially affect NS2B3 proteolytic processing. (A) Schematic of the construction of the triple Ala mutation NS2A/2B-M plasmid. NS2A/2B-M1: the P2, P3 and P4 sites were mutated to Ala; NS2A/2B-M2: the P5, P6 and P7 sites were mutated to Ala; NS2A/2B-M3: the P8, P9 and P10 sites were mutated to Ala. (B) DEFs were cotransfected with NS2A/2B-M and different concentrations of NS2B3, and the cells were harvested 24 h post transfection for WB. Mouse anti-Myc mAb and anti-Flag mAb were used as the primary antibodies.


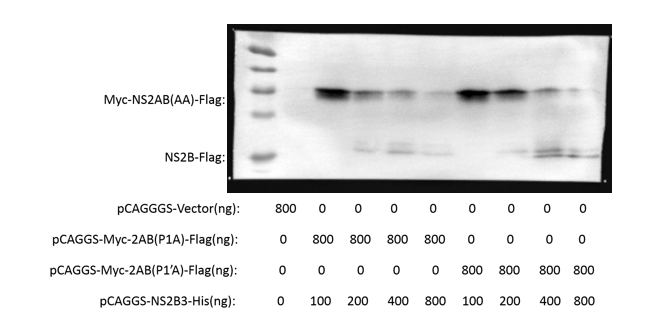


NS2A: 25kDa NS2B: 14kDa EGFP: 25kDa GST: 26kDa EGFP-20aa-GST: 50kDa 2A-20aa-

GST: 49 kDa EGFP-20aa-2B:39 kDa NS2Aβ-GST: 26kDa


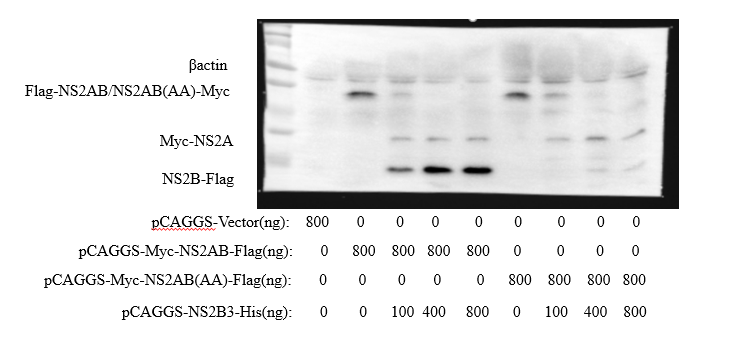


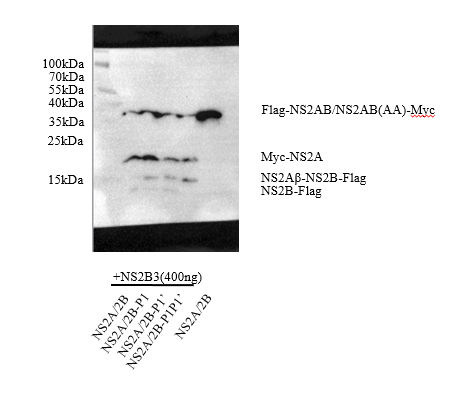


NS2A: 25kDa NS2B: 14kDa EGFP: 25kDa GST: 26kDa EGFP-20aa-GST: 50kDa 2A-20aa-

GST: 49 kDa EGFP-20aa-2B:39 kDa NS2Aβ-GST: 26kDa

Fig. 4. The P1 and P1’ amino acid sites of NS2A/2B are vital for NS2B3 proteolytic processing. (A) Schematic of plasmid construction whereby P1 and/or P1’ were mutated to Ala in NS2A/2B-P. (B, C) DEFs were cotransfected with empty vector or a cleavage construct (NS2A/2B-P1, NS2A/2B-P1’ or NS2A/2B-P1P1’) and different concentrations of NS2B3, and the cells were harvested 24 h post transfection for WB analysis. Mouse anti-Flag monoclonal antibody and mouse anti-Myc monoclonal antibody were used as the primary antibodies simultaneously.
